# The International Medical Annual

**Published:** 1897-02

**Authors:** 


THE INTERNATIONAL MEDICAL ANNUAL, for 1897. A complete work of reference for medical practitioners. The conjoint authorship of forty-one distinguished American, British, and Continental authorities. Has the largest circulation of any medical periodical (not a newspaper) published. E. B. Treat, Publisher, 5 Cooper Union, New York. The Publisher says of it:

"We feel some pride in asserting that no medical work of such a widely international character has been previously issued by the medical press in any country, which offers so much at so small a price. The kind reception and success accorded to the *Annual*, the largeness of which alone, has rendered it possible for us to spare no expense in its production; while the editorial staff have devoted a large amount of time and labor in so condensing the literary matter as to confine the volume within a reasonable size without omitting facts of practical importance.

"We bespeak the continued patronage of the profession that we may thus still further extend the *Annual's* circulation and usefulness."

